# Supplementary material for: Incidence and risk factors for post-stroke delirium in the elderly: A national inpatient sample (NIS) analysis
Source: PLoS One. 2026 Jan 30;21(1):e0331158. doi: 10.1371/journal.pone.0331158 (PMC12857935; doi:10.1371/journal.pone.0331158)
Supplement: S1 File — (PDF) [file pone.0331158.s001.pdf]

S1 File. ICD-9-CM and ICD-10-CM diagnosis codes.

Supplemental Digital Content (Table S1): International Classification of Diseases, 9th Revision, Clinical Modification and Procedure Coding System (ICD-9 CM/PCS) Codes That Were Used.

Diagnosis/ Procedure	ICD-9 CM/PCS code
1. Stroke	433, 43401, 43411, 43491, 431, 432.9
2. Ischemic stroke	433, 43401, 43411, 43491
3. Hemorrhagic stroke	431, 432.9
4. Delirium	290.11, 290.3, 290.41, 291.0, 292.81, 293.0, 293.1, 348.31, 349.82, 780

Supplemental Digital Content (Table S2): International Classification of Diseases, 10th Revision, Clinical Modification and Procedure Coding System (ICD-10 CM/PCS) Codes That Were Used.

Diagnosis/ Procedure	ICD-10 CM/PCS code
1. Stroke	I61, I62.9, I63
2. Ischemic stroke	I63
3. Hemorrhagic stroke	I61, I62.9
4. Delirium	R41.x*, R41.81, F01.51, F02.81, F03.91, F05.x*, F06.x*, F06.2, F06.8, G93.4, G93.49, G93.41, G92.9, G92.8

*Abbreviations: .x: including subcodes, .00-.99
