# Supplementary material for: Incidence and risk factors for post-stroke delirium in the elderly: A national inpatient sample (NIS) analysis
Source: PLoS One. 2026 Jan 30;21(1):e0331158. doi: 10.1371/journal.pone.0331158 (PMC12857935; doi:10.1371/journal.pone.0331158)
Supplement: S1 Table — (DOCX) [file pone.0331158.s002.docx]

**S1 Table. Incidence of delirium following ischemic and hemorrhagic strokes.**

| Characteristics | Ischemic stroke | Hemorrhagic stroke |
| --- | --- | --- |
| No delirium total (n=count) | 1,196,152 | 164,395 |
| Delirium total (n=count) | 278,948 | 53,813 |
| Total incidence (%) | 18.9 | 24.7 |
