# Supplementary material for: Incidence and risk factors for post-stroke delirium in the elderly: A national inpatient sample (NIS) analysis
Source: PLoS One. 2026 Jan 30;21(1):e0331158. doi: 10.1371/journal.pone.0331158 (PMC12857935; doi:10.1371/journal.pone.0331158)
Supplement: S2 Table — (DOCX) [file pone.0331158.s003.docx]

**S2 Table. Multivariable Logistic Regression Analysis of Associations Between Sociodemographic factors and Risk of Delirium Following Ischemic and Hemorrhagic Stroke in Elderly Patients.**

| Variable | | Ischemic stroke | | | Hemorrhagic stroke | | |
| --- | --- | --- | --- | --- | --- | --- | --- |
|  |  | **OR** | **95% CI** | ***p*** | **OR** | **95% CI** | ***p*** |
| Age ≥80 years old | | 1.261 | 1.251-1.272 | <0.001 | 1.093 | 1.071-1.116 | <0.001 |
| Female | | 1.011 | 1.002-1.020 | 0.012 | 0.965 | 0.946-0.984 | <0.001 |
| Race | |  |  |  |  |  |  |
|  | White | Ref | —— | —— | Ref | —— | —— |
|  | Black | 1.115 | 1.100-1.130 | <0.001 | 1.137 | 1.101-1.173 | <0.001 |
|  | Hispanic | 1.003 | 0.985-1.021 | 0.744 | 0.979 | 0.942-1.018 | 0.296 |
|  | Asian or Pacific Islander | 1.029 | 1.001-1.058 | 0.043 | 1.052 | 1.000-1.106 | 0.050 |
|  | Native American | 1.026 | 0.958-1.097 | 0.466 | 1.013 | 0.860-1.193 | 0.874 |
|  | Other | 1.103 | 1.085-1.121 | <0.001 | 1.035 | 0.998-1.074 | 0.067 |
| Number of Comorbidity | |  |  |  |  |  |  |
|  | 0 | Ref | —— | —— | Ref | —— | —— |
|  | 1 | 1.041 | 1.002-1.081 | 0.039 | 1.125 | 1.033-1.225 | 0.007 |
|  | 2 | 1.157 | 1.116-1.200 | <0.001 | 1.198 | 1.104-1.300 | <0.001 |
|  | ≥3 | 2.090 | 2.018-2.165 | <0.001 | 1.991 | 1.841-2.154 | <0.001 |
| Type of insurance | |  |  |  |  |  |  |
|  | Medicare | Ref | —— | —— | Ref | —— | —— |
|  | Medicaid | 1.055 | 1.018-1.093 | 0.003 | 0.958 | 0.888-1.033 | 0.262 |
|  | Private insurance | 0.949 | 0.934-0.965 | <0.001 | 0.968 | 0.933-1.005 | 0.087 |
|  | Self-pay | 0.943 | 0.892-0.998 | 0.042 | 0.965 | 0.869-1.072 | 0.508 |
|  | No charge | 0.786 | 0.624-0.990 | 0.041 | 1.288 | 0.871-1.904 | 0.205 |
|  | Other | 1.103 | 1.063-1.143 | <0.001 | 1.013 | 0.940-1.091 | 0.742 |
| Bed size of hospital | |  |  |  |  |  |  |
|  | Small | Ref | —— | —— | Ref | —— | —— |
|  | Medium | 1.006 | 0.992-1.019 | 0.403 | 1.069 | 1.029-1.109 | 0.001 |
|  | Large | 1.048 | 1.035-1.061 | <0.001 | 1.204 | 1.164-1.246 | <0.001 |
| Elective admission | | 0.678 | 0.668-0.688 | <0.001 | 0.934 | 0.895-0.975 | 0.002 |
| Teaching hospital | | 0.969 | 0.960-0.979 | <0.001 | 0.987 | 0.963-1.013 | 0.324 |
| Urban hospital | | 1.004 | 0.989-1.020 | 0.584 | 1.180 | 1.120-1.244 | <0.001 |
| Region of hospital | |  |  |  |  |  |  |
|  | Northeast | Ref | —— | —— | Ref | —— | —— |
|  | Midwest or North Central | 1.191 | 1.175-1.208 | <0.001 | 1.273 | 1.233-1.314 | <0.001 |
|  | South | 1.251 | 1.236-1.267 | <0.001 | 1.274 | 1.238-1.310 | <0.001 |
|  | West | 1.277 | 1.259-1.296 | <0.001 | 1.396 | 1.351-1.442 | <0.001 |
