# Supplementary material for: Incidence and risk factors for post-stroke delirium in the elderly: A national inpatient sample (NIS) analysis
Source: PLoS One. 2026 Jan 30;21(1):e0331158. doi: 10.1371/journal.pone.0331158 (PMC12857935; doi:10.1371/journal.pone.0331158)
Supplement: S3 Table — (DOCX) [file pone.0331158.s004.docx]

| Variables | Ischemic stroke | | | Hemorrhagic stroke | | | |
| --- | --- | --- | --- | --- | --- | --- | --- |
|  | **OR** | **95% CI** | ***p*** | **OR** | **95% CI** | ***p*** | |
| Comorbidities |  |  |  |  |  |  |  |
| Acquired immune deficiency syndrome | 0.861 | 0.751-0.987 | 0.032 | 0.836 | 0.619-1.130 | 0.244 |  |
| Alcohol abuse | 1.129 | 1.101-1.158 | <0.001 | 1.121 | 1.059-1.186 | <0.001 |  |
| Deficiency anemia | 1.161 | 1.146-1.175 | <0.001 | 1.196 | 1.152-1.241 | <0.001 |  |
| Rheumatoid arthritis/collagen vascular diseases | 1.031 | 1.005-1.057 | 0.019 | 1.025 | 0.958-1.096 | 0.476 |  |
| Chronic blood loss anemia | 0.948 | 0.903-0.994 | 0.028 | 1.125 | 0.973-1.301 | 0.112 |  |
| Congestive heart failure | 1.203 | 1.190-1.216 | <0.001 | 1.150 | 1.119-1.181 | <0.001 |  |
| Coagulopathy | 1.326 | 1.304-1.347 | <0.001 | 1.204 | 1.168-1.242 | <0.001 |  |
| Depression | 1.254 | 1.238-1.271 | <0.001 | 1.113 | 1.077-1.151 | <0.001 |  |
| Diabetes with chronic complications | 1.066 | 1.053-1.079 | <0.001 | 1.085 | 1.053-1.119 | <0.001 |  |
| Drug abuse | 1.356 | 1.294-1.421 | <0.001 | 1.232 | 1.106-1.372 | <0.001 |  |
| Hypothyroidism | 1.023 | 1.012-1.034 | <0.001 | 0.952 | 0.927-0.979 | 0.001 |  |
| Liver disease | 1.046 | 1.014-1.079 | 0.005 | 0.905 | 0.48-0.966 | 0.003 |  |
| Fluid and electrolyte disorders | 1.910 | 1.892-1.927 | <0.001 | 1.732 | 1.697-1.768 | <0.001 |  |
| Psychoses | 1.813 | 1.769-1.857 | <0.001 | 1.448 | 1.358-1.544 | <0.001 |  |
| Pulmonary circulation disorders | 1.022 | 1.002-1.043 | 0.033 | 0.951 | 0.901-1.003 | 0.064 |  |
| Renal failure | 1.072 | 1.061-1.084 | <0.001 | 1.014 | 0.986-1.042 | 0.340 |  |
| Peptic ulcer disease excluding bleeding | 1.089 | 1.019-1.164 | 0.011 | 1.162 | 1.005-1.344 | 0.043 |  |
| Weight loss | 1.710 | 1.684-1.735 | <0.001 | 1.537 | 1.486-1.590 | <0.001 |  |

**S3 Table. Multivariable Logistic Regression Analysis of Associations Between Comorbidities and Risk of Delirium Following Ischemic and Hemorrhagic Stroke in Elderly Patients.**
