# Supplementary material for: Incidence and risk factors for post-stroke delirium in the elderly: A national inpatient sample (NIS) analysis
Source: PLoS One. 2026 Jan 30;21(1):e0331158. doi: 10.1371/journal.pone.0331158 (PMC12857935; doi:10.1371/journal.pone.0331158)
Supplement: S4 Table — (DOCX) [file pone.0331158.s005.docx]

**S4 Table. Multivariable Logistic Regression Analysis of Associations Between Complications and Risk of Delirium Following Ischemic and Hemorrhagic Stroke in Elderly Patients.**

| Complications | Ischemic stroke | | | Hemorrhagic stroke | | |
| --- | --- | --- | --- | --- | --- | --- |
|  | **OR** | **95% CI** | ***p*** | **OR** | **95% CI** | ***p*** |
| Medical complications, n (%) |  |  |  |  |  |  |
| Dysphagia | 1.301 | 1.286-1.317 | <0.001 | 1.305 | 1.272-1.338 | <0.001 |
| Acute myocardial infarction | 1.172 | 1.152-1.192 | <0.001 | 1.203 | 1.145-1.264 | <0.001 |
| Pneumonia | 1.429 | 1.409-1.450 | <0.001 | 1.433 | 1.389-1.479 | <0.001 |
| Urinary tract infection | 1.716 | 1.698-1.735 | <0.001 | 1.432 | 1.396-1.470 | <0.001 |
| Deep vein thrombosis | 1.252 | 1.213-1.291 | <0.001 | 1.243 | 1.175-1.315 | <0.001 |
| Pulmonary embolism | 1.164 | 1.117-1.213 | <0.001 | 0.949 | 0.875-1.029 | 0.205 |
| Sepsis | 2.427 | 2.389-2.466 | <0.001 | 2.014 | 1.940-2.091 | <0.001 |
