# Supplementary material for: Incidence and risk factors for post-stroke delirium in the elderly: A national inpatient sample (NIS) analysis
Source: PLoS One. 2026 Jan 30;21(1):e0331158. doi: 10.1371/journal.pone.0331158 (PMC12857935; doi:10.1371/journal.pone.0331158)
Supplement: S5 Table — (DOCX) [file pone.0331158.s006.docx]

**S5 Table. Diagnosis of Multicollinearity Among Comorbidity and Complication Factors.**

| Variables | Multicollinearity diagnostics | |
| --- | --- | --- |
|  | **VIF** | **Tolerance** |
| Comorbidities |  |  |
| Acquired immune deficiency syndrome | 1.002 | 0.998 |
| Alcohol abuse | 1.020 | 0.981 |
| Deficiency anemia | 1.037 | 0.964 |
| Rheumatoid arthritis/collagen vascular diseases | 1.002 | 0.998 |
| Chronic blood loss anemia | 1.003 | 0.997 |
| Congestive heart failure | 1.072 | 0.933 |
| Coagulopathy | 1.034 | 0.967 |
| Depression | 1.005 | 0.995 |
| Diabetes with chronic complications | 1.081 | 0.925 |
| Drug abuse | 1.011 | 0.990 |
| Hypothyroidism | 1.008 | 0.992 |
| Liver disease | 1.024 | 0.977 |
| Fluid and electrolyte disorders | 1.061 | 0.943 |
| Psychoses | 1.002 | 0.998 |
| Pulmonary circulation disorders | 1.032 | 0.969 |
| Renal failure | 1.124 | 0.889 |
| Peptic ulcer disease excluding bleeding | 1.001 | 0.999 |
| Weight loss | 1.036 | 0.965 |
|  |  |  |
| Medical complications |  |  |
| Dysphagia | 1.012 | 0.988 |
| Acute myocardial infarction | 1.018 | 0.983 |
| Pneumonia | 1.096 | 0.913 |
| Urinary tract infection | 1.031 | 0.969 |
| Deep vein thrombosis | 1.068 | 0.936 |
| Pulmonary embolism | 1.063 | 0.941 |
| Sepsis | 1.117 | 0.895 |
