# Supplementary material for: Incidence and risk factors for post-stroke delirium in the elderly: A national inpatient sample (NIS) analysis
Source: PLoS One. 2026 Jan 30;21(1):e0331158. doi: 10.1371/journal.pone.0331158 (PMC12857935; doi:10.1371/journal.pone.0331158)
Supplement: Attachment — Submitted filename: Response to Reviewers.docx [file pone.0331158.s008.docx]

Response to Reviewers

Manuscript Number: PONE-D-25-43559

Title: **Incidence and risk factors for post-stroke delirium in the elderly: a national inpatient sample (NIS) analysis**

Indicates corresponding author：Ying Gao^1^

^1^ Department of Gastrointestinal Infection, Houjie Clinical College of Guangdong Medical University, Houjie Hospital, Dongguan, Guangdong, 523945, China.

E-mail: 13711837955@163.com	Telephone: 13711837955

Dear Editors and Reviewers,

We appreciate the opportunity to revise our manuscript entitled " Incidence and Risk Factors for Post-Stroke Delirium in the Elderly: A National Inpatient Sample (NIS) Analysis. " We are grateful to your thoughtful and constructive comments, which have significantly improved the quality and clarity of our work and will help guide our future research. Below we provide detailed, point-by-point responses to each your comment. All changes in the manuscript are highlighted in red. Content requiring deletion is indicated with a red strikethrough. In addition to addressing the specific points raised, we have undertaken a comprehensive revision of the entire manuscript to enhance clarity and presentation. In this response letter, your comments are presented in italics, and our corresponding changes and additions to the manuscript are highlighted in red text. The page numbers and line numbers cited in the reply correspond to the locations in the manuscript bearing revision marks. We have tried our best to make all the revisions clear, and we hope that the revised manuscript meets the requirements for publication.

**Reviewer #1:**

**Comment 1:** *Methodological Clarifications: Definition of Delirium: The study identifies delirium based on ICD-9/10 codes, which are prone to undercoding. Please acknowledge this limitation explicitly and discuss how misclassification bias could affect the reported incidence.*

**Response 1:** Thank you for your insightful feedback. First, we acknowledge that the use of ICD-based administrative data is subject to underreporting bias and that identifying delirium solely through diagnostic codes may underestimate its true prevalence. To mitigate this limitation as far as possible, we adopted a comprehensive ICD-9/10 code set for delirium that was systematically compiled from previously published, peer-reviewed NIS and other administrative-database studies [1,2] to enhance the comparability and validity of our findings.

**The revised discussion now reads:**

Importantly, because delirium in our analysis was identified solely through ICD-9 and ICD-10 diagnostic codes, misclassification and undercoding in administrative data likely led to underascertainment of true delirium cases and an underestimation of the observed incidence [3]. **(Page 15, line 232-234)**

References Cited in the Response:

[1] Kumar M, Patil S, Godoy LDC, Kuo CL, Swede H, Kuchel GA, et al. Demand Ischemia as a Predictor of Mortality in Older Patients With Delirium. Front Cardiovasc Med. 2022;9:917252. https://doi.org/10.3389/fcvm.2022.917252 PMID: 35734279

[2] Taha A, Xu H, Ahmed R, Karim A, Meunier J, Paul A, et al. Medical and economic burden of delirium on hospitalization outcomes of acute respiratory failure: A retrospective national cohort. Medicine (Baltimore). 2023;102(2):e32652. https://doi.org/10.1097/MD.0000000000032652 PMID: 36637939

[3] Sheehan KA, Shin S, Hall E, Mak DYF, Lapointe-Shaw L, Tang T, et al. Characterizing medical patients with delirium: a cohort study comparing ICD-10 codes and a validated chart review method. PLoS One. 2024;19(5):e0302888. https://doi.org/10.1371/journal.pone.0302888 PMID: 38739670

**Comment 2:** *Stroke Subtypes: Clarify which ICD codes were used to identify ischemic and hemorrhagic stroke. Consider a sensitivity analysis by stroke type, if feasible.*

**Response 2:** Thank you for your thoughtful comments. We have revised the Discussion and Results (S1 Table in S2 File) to incorporate stroke-subtype sensitivity analyses (stratified multivariable logistic regressions using the same covariate set as the primary models; ICD case definitions are provided in Supplementary Material S1 File). Key points now discussed and linked to Supplementary S2–S4 Tables in S2 File are: (1) delirium incidence is higher after hemorrhagic than ischemic stroke — likely driven by hematoma-related blood-breakdown products, neuroinflammation, and greater exposure to ICU-level precipitating factors; (2) private insurance and elective admission show a protective association in the overall cohort and the ischemic subgroup but not in hemorrhagic patients, which may reflect differential case severity, monitoring, or coding sensitivity; (3) admission to a teaching hospital was associated with lower recorded delirium in ischemic stroke (not in hemorrhagic), plausibly due to organized stroke-unit care and routine delirium prevention/screening; (4) the association between urban hospitals and delirium appeared only for hemorrhagic stroke and is likely influenced by referral/case-mix and ascertainment bias rather than a direct causal effect; and (5) pre-existing hypothyroidism was associated with increased delirium risk in both subgroups, for which we propose biological mechanisms (cerebral perfusion, inflammation/oxidative stress, and reduced cognitive reserve) and recommend closer thyroid monitoring. We also explicitly acknowledge residual confounding, differential ICD coding sensitivity and selection biases as limitations that temper inference.

**The revised objective now reads:(See** **S1-S4 Tables in S2 File.)**

**The revised results now reads:**

PSD occurred in 18.9% of ischemic strokes and 24.7% of hemorrhagic strokes (S1 Table in S2 File).

**(Page 8, line 142-143)**

**The revised discussion now reads:**

The findings also indicate that the incidence of delirium following hemorrhagic stroke is higher than that following ischemic stroke and the overall prevalence of post-stroke delirium, consistent with previous research [1]. Mechanisms plausibly include hematoma-associated release of blood-breakdown products and reactive oxygen species leading to robust neuroinflammation and increased exposure to ICU-related precipitating factors (e.g., mechanical ventilation, sedatives) [2-4].

**(Page 14-15, line 226-230)**

In our subgroup analyses, pre-existing hypothyroidism showed statistically significant associations with post-stroke delirium in both ischemic and hemorrhagic stroke patients. Several plausible biological mechanisms could explain why pre-existing hypothyroidism might increase the risk of delirium after ischemic stroke. Hypothyroid states promote suboptimal cerebral circulation, which increase the likelihood and severity of ischemic cerebral injury; greater ischemic burden and impaired cerebral perfusion reserve may in turn raise the brain’s vulnerability to acute cognitive disturbances such as delirium [5]. In addition, thyroid hormone deficiency is associated with pro-inflammatory and oxidative stress pathways and can impair blood–brain barrier integrity and neuronal resilience, mechanisms implicated in delirium pathogenesis [6]. Finally, hypothyroidism is linked to chronic cognitive impairment and neuropsychiatric symptoms that reduce cognitive reserve and thereby predispose patients to delirium when exposed to the acute physiological stress of ischemic stroke [7]. Therefore, close monitoring of thyroid function may serve as an effective strategy for preventing post-stroke delirium in elderly patients.

**(Page 18, line 295-307)**

Moreover, private insurance and elective admission were associated with lower odds of post-stroke delirium in the overall cohort and among ischemic stroke patients, but not in the hemorrhagic subgroup. Administrative coding (e.g., ICD) is known to have low sensitivity for detecting delirium and may differentially undercapture cases across stroke types and care settings [8]. Thus, any protective effect of private insurance and elective admission may be detectable in less-fatal ischemic strokes but obscured in the clinically more severe hemorrhagic cohort [9,10]. Residual confounding effects from baseline stroke severity and other unmeasured factors may also contribute.

**(Page 19-20, line 326-332)**

In subgroup analyses by stroke type, hospital characteristics showed divergent associations with post-stroke delirium. We observed that admission to a teaching hospital was associated with lower odds of post-stroke delirium in ischemic stroke patients, but this association was not significant in the overall cohort or the hemorrhagic subgroup. Teaching hospital and certified stroke centers typically deliver organized stroke-unit care, multidisciplinary pathways, and rapid reperfusion, and they more often implement routine delirium screening and prevention, factors that likely account for the reduced recorded delirium in the ischemic subgroup [11]. Additionally, we found that admission to an urban hospital was associated with delirium only among patients with hemorrhagic stroke. Urban centers often care for patients with a higher burden of comorbidity and adverse social determinants of health, both independently linked to delirium, which may partially explain the observed association [12]. In addition, referral and ascertainment biases, whereby severe hemorrhagic stroke cases are preferentially transferred to tertiary urban hospitals and undergo more intensive monitoring, could amplify the association in the hemorrhagic subgroup relative to ischemic stroke [12]. Thus, the higher delirium prevalence after hemorrhagic stroke seen in urban hospitals likely reflects a combination of greater baseline severity, ICU-related risks, and differential case ascertainment rather than a direct causal effect of hospital location.

**(Page 20, line 336-351)**

References Cited in the Response:

[1] Zhang G-B, Li H-Y, Yu W-J, Ying Y-Z, Zheng D, Zhang X-K, et al. Occurrence and risk factors for post-stroke delirium: a systematic review and m-eta-analysis. Asian J Psychiatry. 2024;99:104132. https://doi.org/10.1016/j.ajp.2024.104132 PMID: 38981150

[2] Zhang G-B, Lv J-M, Yu W-J, Li H-Y, Wu L, Zhang S-L, et al. The associations of post-stroke delirium with outcomes: A systematic review and me-ta-analysis. BMC Med. 2024;22(1):470. https://doi.org/10.1186/s12916-024-03689-1 PMID: 39407191

[3] Fan Y-Y, Luo R-Y, Wang M-T, Yuan C-Y, Sun Y-Y, Jing J-Y. Mechanisms underlying delirium in patients with critical illness. Front Aging Neurosci. 2024;16. https://doi.org/10.3389/fnagi.2024.1446523 PMID: 39391586

[4] Krishnan K, Campos PB, Nguyen TN, Tan CW, Chan SL, Appleton JP, et al. Cerebral edema in intracerebral hemorrhage: pathogenesis, natural history, and potential treatments from translation to clinical trials. Front Stroke. 2023;2. https://doi.org/10.3389/fstro.2023.1256664

[5] Tian Y, Shi XQ, Shui JW, Liu XY, Bu Y, Liu Y, et al. Exploring the cau-sal factor effects of hypothyroidism on ischemic stroke: A two-sample men-delian randomization study. Front Neurol. 2024;15. https://doi.org/10.3389/fneur.2024.1322472 PMID: 38361639

[6] Zúñiga D, Balasubramanian S, Mehmood KT, Al-Baldawi S, Zúñiga Salazar G. Hypothyroidism and cardiovascular disease: a review. Cureus. 2024;16(1):e52512. https://doi.org/10.7759/cureus.52512 PMID: 38370998

[7] Sheng X, Gao J, Chen K, Zhu X, Wang Y. Hyperthyroidism, hypothyroidis-m, thyroid stimulating hormone, and dementia risk: Results from the NHA-NES 2011–2012 and mendelian randomization analysis. Front Aging Neurosci. 2024;16. https://doi.org/10.3389/fnagi.2024.1456525 PMID: 39507203

[8] Sheehan KA, Shin S, Hall E, Mak DYF, Lapointe-Shaw L, Tang T, et al. Characterizing medical patients with delirium: a cohort study comparing ICD-10 codes and a validated chart review method. PLoS One. 2024;19(5):e0302888. https://doi.org/10.1371/journal.pone.0302888 PMID: 38739670

[9] Nguyen MTH, Sakamoto Y, Maeda T, Woodward M, Anderson CS, Catiwa J, et al. Influence of socioeconomic status on functional outcomes after stroke: a systematic review and meta-analysis. J Am Heart Assoc. 2024;13(9):e033078. https://doi.org/10.1161/JAHA.123.033078 PMID: 38639361

[10] Dagli C, Huang Z, Lin C. Intracerebral hemorrhage hospitalizations and outcomes: Comparisons between institutional and national data. Intern Emerg Med. 2025;20(5):1455–1462. https://doi.org/10.1007/s11739-025-03977-5 PMID: 40437327

[11] Shen Y-C, Kim AS, Hsia RY. Treatments and patient outcomes following s-troke center expansion. JAMA Netw Open. 2024;7(11):e2444683. https://doi.org/10.1001/jamanetworkopen.2024.44683 PMID: 39535793

[12] Loggini A, Hornik J, Schwertman A, Hornik A. Rural-urban disparities in acute stroke treatments and outcomes: A propensity score-matched analysis of a nationwide sample. Cerebrovasc Dis. 2025;1-9. https://doi.org/10.1159/000546950 PMID: 40562010

**Comment 3:** *Multicollinearity: Explain how potential overlap between comorbidities (e.g., sepsis, metabolic disorders, infection) was addressed in regression modeling.*

**Response 3:** Thank you for raising this important point. In the submitted revision we have already performed VIF-based collinearity diagnostics and report that all independent variables returned VIF values below 5. The results of diagnosing multicollinearity among comorbidity and complication factors are detailed in S5 Table of S2 File.

**The revised method now reads:**

Univariate and multivariate logistic regression analyses were performed to identify independent risk factors for post-stroke delirium; before fitting the multivariate models, multicollinearity diagnostics were conducted separately for the two predictor categories (comorbidities and complications) [1].

**(Page 7, line 130-133)**

**The revised result now reads:**

Collinearity was assessed using variance inflation factors; all independent variables returned VIF values below 5, indicating that multicollinearity was not a concern (S5 Table in S2 File) [1].

**(Page 10, line 172-173)**

References Cited in the Response:

[1] Marcoulides KM, Raykov T. Evaluation of variance inflation factors in regr-ession models using latent variable modeling methods. Educ Psychol Meas. 2019;79(5):874–882. https://doi.org/10.1177/0013164418817803 PMID: 31488917

**Comment 4:** *Interpretation and Discussion: Some predictors (e.g., race, elective admission) have small odds ratios but are statistically significant. Discuss clinical vs. statistical significance and interpret findings within the context of real-world relevance.*

**Response 4:**

Thank you for this important point. We fully agree that statistical significance does not necessarily imply clinical importance, particularly for analyses of large administrative datasets such as the NIS. In response to your recommendation we have revised the Discussion to explicitly distinguish statistical from clinical significance for the protective factors (private insurance and elective admission) as well as for race, and to clarify plausible mechanisms and limitations.

**The revised discussion now reads:**

Although these associations reached statistical significance, the magnitude of the effects was small. Moreover, because our analysis is based on a large administrative dataset (the National Inpatient Sample, NIS), the substantial sample size can make modest between-group differences statistically significant without implying substantial clinical impact [1]. Possible explanations for the observed disparities include socioeconomic and healthcare access differences, cultural factors influencing symptom recognition and help-seeking, and unmeasured clinical or social confounders; in addition, we cannot rule out the possibility that racial differences in diagnostic coding or in the identification of delirium within administrative data may have influenced the observed associations. Clinically, while the modest effect sizes temper claims about a large individual-level impact, these findings nonetheless identify patient groups who may warrant closer clinical monitoring and tailored delirium-prevention interventions [2]. Further work using richer clinical data is warranted to clarify causal pathways and to estimate absolute risk increases in more clinically granular cohorts.

**(Page 15-16, line 243-255)**

Elective admission also implies lower baseline disease severity and greater opportunities for pre-admission optimization, such as medication reconciliation, correction of metabolic abnormalities, treatment of concurrent infections, and patient/caregiver education [3,4]. Consequently, compared to unplanned emergency admissions, elective admissions carry lower short-term physiological vulnerability to delirium [3]. Although both associations were statistically significant, the effect sizes were modest, and the large NIS sample may have amplified small between-group differences without clear clinical significance at the individual level [1].

**(Page 19, line 319-326)**

References Cited in the Response:

[1] Li J, Li Q, Zhang J, Chen X, Yang L, Zhang Y, et al. Incidence, outcomes and risk factors of postoperative urinary retention in patients undergoing primary total knee arthroplasty: A national inpatient sample database study. BMC Surg. 2025;25(1):186. https://doi.org/10.1186/s12893-025-02930-9 PMID: 40296087.

[2] Filler J, Georgakis MK, Dichgans M. Risk factors for cognitive impairment and dementia after stroke: a systematic review and meta-analysis. Lancet, Healthy Longev. 2024;5(1):e31–e44. https://doi.org/10.1016/S2666-7568(23)00217-9 PMID: 38101426

[3] Zeoli T, Chanbour H, Ahluwalia R, Abtahi AM, Stephens BF, Zuckerman SL. Does elective admission vs. Emergency department presentation affect surgical outcomes in metastatic spine surgery? Diagn (Basel Switz). 2024;14(10):1058. https://doi.org/10.3390/diagnostics14101058 PMID: 38786356

[4] Yamada Y, Kobayashi R, Yamamoto T, Fujii H, Iihara H, Hiroko K-H, et al. Medication reconciliation by pharmacists for pre-admission patients improves patient safety. J Pharm Health Care Sci. 2024;10:19–28. https://doi.org/10.1186/s40780-024-00340-2 PMID: 38671535

**Comment 5:** *Expand discussion on protective factors (e.g., private insurance, elective admission) — likely reflecting socioeconomic and health status differences.*

**Response 5:** Thank you for the constructive suggestion to expand the Discussion regarding protective factors (private insurance and elective admission). We have expanded the discussion to elaborate on how private insurance and elective admission serve as protective factors against post-stroke delirium, likely as proxies for socioeconomic advantages and better baseline health optimization. We have incorporated supporting evidence from recent literature to strengthen the interpretation.

**The revised discussion now reads:**

These associations are more likely to reflect broader socioeconomic disparities and baseline health differences. Research indicates that lack of private insurance or other indicators of social disadvantage are independently associated with an increased risk of delirium, and that social determinants including healthcare accessibility, caregiver support, and community disadvantage play an important role in delirium susceptibility [1,2]. Among older adults, private insurance typically represents greater economic resources, improved access to primary and specialty care, and enhanced availability of social support and caregiver assistance, the latter facilitates medication adherence, follow-up management, and early recognition of clinical changes [3]. These advantages contribute to improved chronic disease control (e.g., heart failure, diabetes) and more timely management of acute precipitating factors (e.g., infection, electrolyte imbalance), collectively reducing the risk of delirium in frail stroke patients [4-6]. Elective admission also implies lower baseline disease severity and greater opportunities for pre-admission optimization, such as medication reconciliation, correction of metabolic abnormalities, treatment of concurrent infections, and patient/caregiver education [7,8]. Consequently, compared to unplanned emergency admissions, elective admissions carry lower short-term physiological vulnerability to delirium [7].

**(Page 18-19, line 309-323)**

References Cited in the Response:

[1] Nguyen MTH, Sakamoto Y, Maeda T, Woodward M, Anderson CS, Catiwa J, et al. Influence of socioeconomic status on functional outcomes after stroke: a systematic review and meta-analysis. J Am Heart Assoc. 2024;13(9):e033078. https://doi.org/10.1161/JAHA.123.033078 PMID: 38639361

[2] Arias F, Dufour AB, Jones RN, Alegria M, Fong TG, Inouye SK. Social determinants of health and incident postoperative delirium: exploring key rel-ationships in the SAGES study. J Am Geriatr Soc. 2024;72(2):369–381. https://doi.org/10.1111/jgs.18662 PMID: 37933703

[3] Zachrison KS, Hsia RY, Schwamm LH, Yan Z, Samuels-Kalow ME, Reeves MJ, et al. Insurance-based disparities in stroke center access in california: a network science approach. Circ: Cardiovasc Qual Outcomes. 2023;16:e009868. https://doi.org/10.1161/CIRCOUTCOMES.122.009868 PMID: 37746725

[4] Fan Y-Y, Luo R-Y, Wang M-T, Yuan C-Y, Sun Y-Y, Jing J-Y. Mechanisms underlying delirium in patients with critical illness. Front Aging Neurosci. 2024;16. https://doi.org/10.3389/fnagi.2024.1446523 PMID: 39391586

[5] Wang W, Zhang Y, Yao W, Tang W, Li Y, Sun H, et al. Association betw-een preoperative persistent hyperglycemia and postoperative delirium in ger-iatric hip fracture patients. BMC Geriatr. 2024;24(1):585–596. https://doi.org/10.1186/s12877-024-05192-x PMID: 38977983

[6] Orkaby AR, Hshieh TT. Between a rock and a hard place: risks of deliriu-m and frailty in heart failure patients. JACC Adv. 2024;3(10):101273. https://doi.org/10.1016/j.jacadv.2024.101273 PMID: 39345901

[7] Zeoli T, Chanbour H, Ahluwalia R, Abtahi AM, Stephens BF, Zuckerman SL. Does elective admission vs. Emergency department presentation affect s-urgical outcomes in metastatic spine surgery? Diagn (Basel Switz). 2024;14(10):1058. https://doi.org/10.3390/diagnostics14101058 PMID: 38786356

[8] Yamada Y, Kobayashi R, Yamamoto T, Fujii H, Iihara H, Hiroko K-H, et al. Medication reconciliation by pharmacists for pre-admission patients impr-oves patient safety. J Pharm Health Care Sci. 2024;10:19–28. https://doi.org/10.1186/s40780-024-00340-2 PMID: 38671535

**Comment 6:** *Highlight that delirium identification may be influenced by diagnostic vigilance bias — patients in tertiary centers may have higher detection rates.*

**Response 6:** We appreciate your valuable suggestion and have revised the discussion to explicitly highlight the potential influence of diagnostic vigilance bias on delirium identification, particularly in tertiary centers where detection rates may be higher due to advanced resources and specialized staff. We have incorporated supporting evidence from recent literature to strengthen the interpretation.

**The revised discussion now reads:**

In addition to patient-related physiological factors, hospital bed capacity was independently associated with post-stroke delirium in our logistic regression analysis. Notably, hospitals with greater bed sizes (detailed specifications for bed sizes are available at the following URL: https://hcup-us.ahrq.gov/db/nation/nis/nisdbdocumentation.jsp) exhibited the highest incidence of delirium, which may reflect ascertainment bias arising from heightened diagnostic vigilance that enhances delirium detection [1]. Specifically, such hospitals are typically large tertiary care centers equipped with advanced medical technologies and staffed by highly specialized personnel, thereby contributing to elevated detection rates [1,2].

**(Page 17, line 270-277)**

**The revised limitations now reads:**

Furthermore, the lack of standardized delirium screening protocols in this study, together with potential diagnostic alertness bias arising from heterogeneity in hospital settings (e.g., higher detection rates in tertiary care facilities), may contribute to discrepancies between reported post-stroke delirium incidence and its true prevalence.

**(Page 22, line 388-391)**

References Cited in the Response:

[1] Boucher EL, Gan JM, Lovett NG, Smith SC, Shepperd S, Pendlebury ST. Delirium prevalence, diagnostic uncertainty and outcomes in ORCHARD-EPR: validation against prospective reference cohorts. Age Ageing. 2025;54:afaf284. https://doi.org/10.1093/ageing/afaf284 PMID: 41100407

[2] Lindroth H, Liu K, Szalacha L, Ashkenazy S, Bellelli G, van den Boogaard M, et al. World delirium awareness and quality survey in 2023—a worldwide point prevalence study. Age Ageing. 2024;53(11):afae248. https://doi.org/10.1093/ageing/afae248 PMID: 39568389

**Comment 7:** *Presentation and Organization: Abstract: Include the most impactful predictors with adjusted odds ratios to strengthen clinical takeaways.*

**Response 7:** Thank you for your constructive suggestions. We have revised the abstract to report major predictors and their odds ratios (OR) in the multivariate model. This approach highlights the associations with the greatest clinical relevance while fully preserving the multivariate estimates (OR、95% confidence intervals and p-value) in the results section and tables.

**The revised abstract now reads:**

Major risk factors for PSD include: sepsis (OR = 2.364, 95%CI = 2.329-2.400)、three or more comorbidities (OR = 2.049, 95%CI = 1.984-2.116) and fluid/electrolyte disorders (OR = 1.902, 95%CI = 1.886-1.918)、psychoses (OR = 1.765, 95%CI = 1.725-1.806)、weight loss (OR = 1.690, 95%CI = 1.667-1.714)、urinary tract infection (OR = 1.685, 95%CI = 1.668-1.702).

**(Page 3, line 49-52)**

**Comment 8:** *Tables/Figures: Consider combining redundant figures and adding a forest plot summarizing major risk factors.*

**Response 8:** Thank you for this insightful comment. Considering that the results section and Table 2 already present the demographic and hospital characteristics of patients in the delirium and non-delirium groups, we removed the original pie chart comparing these characteristics between groups to avoid redundancy. To better illustrate the primary risk factors, we created a forest plot for risk factors with OR≥1.20 that were statistically significant, as shown in Fig 2 in the main text.

**The revised objective now reads:(See Fig 2 for details.)**

**Comment 9:** *Language and Grammar: Minor copyediting suggested for precision and readability. Example: “Patients in the delirium group exhibited a higher proportion of patients aged 80 years and above” → “The delirium group had a greater proportion of patients aged ≥80 years.”*

**Response 9:** We greatly appreciate your constructive feedback. We have revised the text where language was insufficient, following the examples provided in your comments, to enhance the accuracy and readability of the article.

**The revised manuscript** **now reads:**

The delirium group had a greater proportion of patients aged ≥80 years (43.5% vs. 43.2%, *p* < 0.001).

**(Page 8, line 146-147)**

Logistic regression analysis indicated that individuals aged ≥80 years had a significantly higher likelihood of developing delirium (Table 3).

**(Page 16, line 260-261)**

Furthermore, the likelihood of delirium was elevated in patients presenting with ≥3 comorbidities (Table 3).

**(Page 16, line 265-266)**

**Comment 10:** *Literature and References: Some references are duplicated (e.g., 8 and 13). Please revise for consistency.*

**Response 10:** Thank you for the careful reading and for pointing out the issue with duplicated references. We have addressed this concern thoroughly. Specifically, we identified and consolidated duplicated entries (for example, references originally listed as Nos. 8 and 13 referred to the same source) into single, unique citations. Merged duplicate reference entries and updated all corresponding in-text citations so each source appears only once in the reference list. Renumbered the reference list and verified that every in-text citation matches the revised reference numbering. Standardized all reference entries to comply with PLOS ONE formatting requirements and verified bibliographic details to correct any incomplete or inconsistent records. Performed a final cross-check of the entire bibliography to ensure no remaining duplicates or mismatches. We appreciate the suggestion and believe these changes improve the manuscript’s clarity and accuracy.

**Comment 11:** *Incorporate recent literature (2023–2024) on delirium prediction and prevention after stroke.*

**Response 11:** Thank you for your insightful feedback. In line with your suggestion, we have reviewed and incorporated recent literature (2023–2024) on post-stroke delirium, and have integrated key findings into the relevant sections of the revised manuscript to strengthen the discussion of incidence and risk factors and to ensure that the citations reflect the current state of the field. The changes to the references are as follows (Detailed changes to the references are available in the revised draft with revision marks):

**Previous References:**

van der Wulp K, van Wely M, van Heijningen L, van Bakel B, Schoon Y, Verkroost M, Gehlmann H, Van Garsse L, Vart P, Kievit P, Rikkert MO, Morshuis W, van Royen N. Delirium After Transcatheter Aortic Valve Implantation Under General Anesthesia: Incidence, Predictors, and Relation to Long-Term Survival. J Am Geriatr Soc. 2019 Nov;67(11):2325-2330.

**Replaced References:**

Ye F, Ho MH, Lee JJ. Prevalence of post-stroke delirium in acute settings: A systematic review and meta-analysis. Int J Nurs Stud. 2024 Jun;154:104750. doi: 10.1016/j.ijnurstu.2024.104750. PMID: 38552472.

**Reviewer #2:**

**Comment 1:** *Being a retrospective Design, it limits diagnostic accuracy of delirium and the assessment of influencing risk factors.*

**Response 1:** Thank you for your constructive feedback. We acknowledge that, as a retrospective study using ICD codes to identify delirium, misclassification and underreporting are possible and may have affected case ascertainment and the estimated associations; these issues are acknowledged and discussed in the Limitations section. To reduce this bias, we employed an exhaustive ICD-9/ICD-10 code list for delirium that was systematically compiled from prior peer-reviewed NIS and administrative-database studies [1,2], thereby improving case ascertainment and comparability. Nevertheless, the study retains several important strengths, 1. Large, nationally representative sample: Use of the NIS provides substantial statistical power and enhances generalizability across diverse geographic and demographic populations; 2. High clinical relevance: The study focuses on post-stroke delirium, a common yet under-recognized complication with important implications for patient outcomes and healthcare costs; 3. Comprehensive risk factor assessment: Both modifiable and non-modifiable risk factors are systematically evaluated; 4. Robust analytic approach: Multivariate logistic regression with appropriate adjustment for confounders ensures the reliability of the findings; 5. Clear presentation: Results are well organized and clearly presented through concise tables and figures, facilitating reader comprehension.

**The limitation now reads:**

The retrospective design of this study limits our capacity to infer causal relationships, while the reliance on administrative data may have led to underreporting or inaccuracies in the coding of variables such as delirium, thereby potentially underestimating the incidence of post-stroke delirium. **(Page 22, line 387-390)**

References Cited in the Response:

[1] Kumar M, Patil S, Godoy LDC, Kuo CL, Swede H, Kuchel GA, et al. Demand Ischemia as a Predictor of Mortality in Older Patients With Delirium. Front Cardiovasc Med. 2022;9:917252. https://doi.org/10.3389/fcvm.2022.917252 PMID: 35734279

[2] Taha A, Xu H, Ahmed R, Karim A, Meunier J, Paul A, et al. Medical and economic burden of delirium on hospitalization outcomes of acute respiratory failure: A retrospective national cohort. Medicine (Baltimore). 2023;102(2):e32652. https://doi.org/10.1097/MD.0000000000032652 PMID: 36637939

**Comment 2:** *Severity and subtype of stroke are not specified, which could significantly affect outcomes.*

**Response 2:** Thank you for this important observation. We agree that stroke severity and subtype can materially influence the risk of post-stroke delirium. Because the National Inpatient Sample does not contain a standardized stroke severity score, direct adjustment for severity was not possible; we have acknowledged this as a limitation in the revised manuscript. To address your concern about heterogeneity by stroke type, we re-extracted the data to classify strokes by subtype using diagnosis codes and performed separate multivariable logistic regression analyses for ischemic and hemorrhagic stroke cohorts. These new subtype-specific analyses are summarized in S2–S4 Tables of S2 File (Supplementary Materials). Overall, the principal risk factors identified in the pooled analysis remained robust, while a small number of predictors showed subtype-specific differences; we now describe these differences and their possible clinical implications in the revised Discussion. (1) delirium incidence is higher after hemorrhagic than ischemic stroke — likely driven by hematoma-related blood-breakdown products, neuroinflammation, and greater exposure to ICU-level precipitating factors; (2) private insurance and elective admission show a protective association in the overall cohort and the ischemic subgroup but not in hemorrhagic patients, which may reflect differential case severity, monitoring, or coding sensitivity; (3) admission to a teaching hospital was associated with lower recorded delirium in ischemic stroke (not in hemorrhagic), plausibly due to organized stroke-unit care and routine delirium prevention/screening; (4) the association between urban hospitals and delirium appeared only for hemorrhagic stroke and is likely influenced by referral/case-mix and ascertainment bias rather than a direct causal effect; and (5) pre-existing hypothyroidism was associated with increased delirium risk in both subgroups, for which we propose biological mechanisms (cerebral perfusion, inflammation/oxidative stress, and reduced cognitive reserve) and recommend closer thyroid monitoring. We appreciate the your suggestion, which has strengthened the manuscript’ s transparency and interpretability.

**The revised objective now reads:(See** **S1-S4 Tables in S2 File.)**

**The revised results now reads:**

PSD occurred in 18.9% of ischemic strokes and 24.7% of hemorrhagic strokes (S1 Table in S2 File).

**(Page 8, line 142-143)**

**The revised discussion now reads:**

The findings also indicate that the incidence of delirium following hemorrhagic stroke is higher than that following ischemic stroke and the overall prevalence of post-stroke delirium, consistent with previous research [1]. Mechanisms plausibly include hematoma-associated release of blood-breakdown products and reactive oxygen species leading to robust neuroinflammation and increased exposure to ICU-related precipitating factors (e.g., mechanical ventilation, sedatives) [2-4].

**(Page 14-15, line 226-230)**

In our subgroup analyses, pre-existing hypothyroidism showed statistically significant associations with post-stroke delirium in both ischemic and hemorrhagic stroke patients. Several plausible biological mechanisms could explain why pre-existing hypothyroidism might increase the risk of delirium after ischemic stroke. Hypothyroid states promote suboptimal cerebral circulation, which increase the likelihood and severity of ischemic cerebral injury; greater ischemic burden and impaired cerebral perfusion reserve may in turn raise the brain’s vulnerability to acute cognitive disturbances such as delirium [5]. In addition, thyroid hormone deficiency is associated with pro-inflammatory and oxidative stress pathways and can impair blood–brain barrier integrity and neuronal resilience, mechanisms implicated in delirium pathogenesis [6]. Finally, hypothyroidism is linked to chronic cognitive impairment and neuropsychiatric symptoms that reduce cognitive reserve and thereby predispose patients to delirium when exposed to the acute physiological stress of ischemic stroke [7]. Therefore, close monitoring of thyroid function may serve as an effective strategy for preventing post-stroke delirium in elderly patients.

**(Page 18, line 295-307)**

Moreover, private insurance and elective admission were associated with lower odds of post-stroke delirium in the overall cohort and among ischemic stroke patients, but not in the hemorrhagic subgroup. Administrative coding (e.g., ICD) is known to have low sensitivity for detecting delirium and may differentially undercapture cases across stroke types and care settings [8]. Thus, any protective effect of private insurance and elective admission may be detectable in less-fatal ischemic strokes but obscured in the clinically more severe hemorrhagic cohort [9,10]. Residual confounding effects from baseline stroke severity and other unmeasured factors may also contribute.

**(Page 19-20, line 326-332)**

In subgroup analyses by stroke type, hospital characteristics showed divergent associations with post-stroke delirium. We observed that admission to a teaching hospital was associated with lower odds of post-stroke delirium in ischemic stroke patients, but this association was not significant in the overall cohort or the hemorrhagic subgroup. Teaching hospital and certified stroke centers typically deliver organized stroke-unit care, multidisciplinary pathways, and rapid reperfusion, and they more often implement routine delirium screening and prevention, factors that likely account for the reduced recorded delirium in the ischemic subgroup [11]. Additionally, we found that admission to an urban hospital was associated with delirium only among patients with hemorrhagic stroke. Urban centers often care for patients with a higher burden of comorbidity and adverse social determinants of health, both independently linked to delirium, which may partially explain the observed association [12]. In addition, referral and ascertainment biases, whereby severe hemorrhagic stroke cases are preferentially transferred to tertiary urban hospitals and undergo more intensive monitoring, could amplify the association in the hemorrhagic subgroup relative to ischemic stroke [12]. Thus, the higher delirium prevalence after hemorrhagic stroke seen in urban hospitals likely reflects a combination of greater baseline severity, ICU-related risks, and differential case ascertainment rather than a direct causal effect of hospital location.

**(Page 20, line 336-351)**

**The revised limitations now reads:**

Meanwhile, the lack of data in the database regarding key risk factors for delirium, notably stroke severity、pre-stroke cognitive status、drug type and frequency of administration, may have limited the precision of our estimates.

**(Page 23, line 394-396)**

References Cited in the Response:

[1] Zhang G-B, Li H-Y, Yu W-J, Ying Y-Z, Zheng D, Zhang X-K, et al. Occurrence and risk factors for post-stroke delirium: a systematic review and m-eta-analysis. Asian J Psychiatry. 2024;99:104132. https://doi.org/10.1016/j.ajp.2024.104132 PMID: 38981150

[2] Zhang G-B, Lv J-M, Yu W-J, Li H-Y, Wu L, Zhang S-L, et al. The associations of post-stroke delirium with outcomes: A systematic review and me-ta-analysis. BMC Med. 2024;22(1):470. https://doi.org/10.1186/s12916-024-03689-1 PMID: 39407191

[3] Fan Y-Y, Luo R-Y, Wang M-T, Yuan C-Y, Sun Y-Y, Jing J-Y. Mechanisms underlying delirium in patients with critical illness. Front Aging Neurosci. 2024;16. https://doi.org/10.3389/fnagi.2024.1446523 PMID: 39391586

[4] Krishnan K, Campos PB, Nguyen TN, Tan CW, Chan SL, Appleton JP, et al. Cerebral edema in intracerebral hemorrhage: pathogenesis, natural history, and potential treatments from translation to clinical trials. Front Stroke. 2023;2. https://doi.org/10.3389/fstro.2023.1256664

[5] Tian Y, Shi XQ, Shui JW, Liu XY, Bu Y, Liu Y, et al. Exploring the cau-sal factor effects of hypothyroidism on ischemic stroke: A two-sample men-delian randomization study. Front Neurol. 2024;15. https://doi.org/10.3389/fneur.2024.1322472 PMID: 38361639

[6] Zúñiga D, Balasubramanian S, Mehmood KT, Al-Baldawi S, Zúñiga Salazar G. Hypothyroidism and cardiovascular disease: a review. Cureus. 2024;16(1):e52512. https://doi.org/10.7759/cureus.52512 PMID: 38370998

[7] Sheng X, Gao J, Chen K, Zhu X, Wang Y. Hyperthyroidism, hypothyroidis-m, thyroid stimulating hormone, and dementia risk: Results from the NHA-NES 2011–2012 and mendelian randomization analysis. Front Aging Neurosci. 2024;16. https://doi.org/10.3389/fnagi.2024.1456525 PMID: 39507203

[8] Sheehan KA, Shin S, Hall E, Mak DYF, Lapointe-Shaw L, Tang T, et al. Characterizing medical patients with delirium: a cohort study comparing ICD-10 codes and a validated chart review method. PLoS One. 2024;19(5):e0302888. https://doi.org/10.1371/journal.pone.0302888 PMID: 38739670

[9] Nguyen MTH, Sakamoto Y, Maeda T, Woodward M, Anderson CS, Catiwa J, et al. Influence of socioeconomic status on functional outcomes after stroke: a systematic review and meta-analysis. J Am Heart Assoc. 2024;13(9):e033078. https://doi.org/10.1161/JAHA.123.033078 PMID: 38639361

[10] Dagli C, Huang Z, Lin C. Intracerebral hemorrhage hospitalizations and outcomes: Comparisons between institutional and national data. Intern Emerg Med. 2025;20(5):1455–1462. https://doi.org/10.1007/s11739-025-03977-5 PMID: 40437327

[11] Shen Y-C, Kim AS, Hsia RY. Treatments and patient outcomes following s-troke center expansion. JAMA Netw Open. 2024;7(11):e2444683. https://doi.org/10.1001/jamanetworkopen.2024.44683 PMID: 39535793

[12] Loggini A, Hornik J, Schwertman A, Hornik A. Rural-urban disparities in acute stroke treatments and outcomes: A propensity score-matched analysis of a nationwide sample. Cerebrovasc Dis. 2025;1-9. https://doi.org/10.1159/000546950 PMID: 40562010

**Comment 3:** *Bed size is unclear, and teaching status is mentioned without explaining its impact.*

**Response 3:** Thank you for this helpful comment. We have addressed both points in the revised manuscript: First, we provided the official HCUP/NIS reference URL in the manuscript for readers who wish to verify the classification criteria. In addition, we have expanded the Discussion to explain the observed association between teaching hospitals and delirium. These additions are intended to help readers interpret the observed association and to clarify that the relationship may reflect both true clinical differences and variation in detection or coding.

**The revised discussion now reads:**

Notably, hospitals with greater bed sizes (detailed specifications for bed sizes are available at the following URL: https://hcup-us.ahrq.gov/db/nation/nis/nisdbdocumentation.jsp) exhibited the highest incidence of delirium, which may reflect ascertainment bias arising from heightened diagnostic vigilance that enhances delirium detection [1].

**(Page 17, line 272-275)**

In subgroup analyses by stroke type, hospital characteristics showed divergent associations with post-stroke delirium. We observed that admission to a teaching hospital was associated with lower odds of post-stroke delirium in ischemic stroke patients, but this association was not significant in the overall cohort or the hemorrhagic subgroup. Teaching hospital and certified stroke centers typically deliver organized stroke-unit care, multidisciplinary pathways, and rapid reperfusion, and they more often implement routine delirium screening and prevention, factors that likely account for the reduced recorded delirium in the ischemic subgroup [2].

**(Page 20, line 336-342)**

References Cited in the Response:

[1] Boucher EL, Gan JM, Lovett NG, Smith SC, Shepperd S, Pendlebury ST. Delirium prevalence, diagnostic uncertainty and outcomes in ORCHARD-EPR: validation against prospective reference cohorts. Age Ageing. 2025;54:afaf284. https://doi.org/10.1093/ageing/afaf284 PMID: 41100407

[2] Shen Y-C, Kim AS, Hsia RY. Treatments and patient outcomes following s-troke center expansion. JAMA Netw Open. 2024;7(11):e2444683. https://doi.org/10.1001/jamanetworkopen.2024.44683 PMID: 39535793

**Comment 4:** *It’s unclear whether delirium led to complications (e.g., dysphagia, pneumonia, UTI) or vice versa.*

**Response 4:** Thank you for this constructive comment. We fully acknowledge that, due to the retrospective and observational nature of the NIS database, our study cannot establish a causal relationship or definitively determine the temporal direction between post-stroke delirium and related complications such as dysphagia, pneumonia, urinary tract infection, or sepsis. The associations observed in our analyses should therefore be interpreted as correlational rather than causal. To address this limitation and clarify the potential directionality, we have substantially revised the Discussion section. Specifically, we now integrate evidence from prior cohort studies, experimental research, and mechanistic literature to contextualize our findings. The revised Discussion highlights that certain complications (e.g., dysphagia, pneumonia, and sepsis) may plausibly precede and contribute to delirium through pathways involving malnutrition, systemic inflammation, blood–brain barrier disruption, and neuroinflammation. At the same time, we explicitly note that delirium itself may increase susceptibility to some complications, particularly urinary tract infection, through secondary effects such as dehydration, urinary retention, and increased exposure to indwelling catheters, suggesting a possible bidirectional relationship. Finally, we emphasize the need for large, high-quality prospective studies to disentangle temporal sequencing and causal pathways between post-stroke delirium and its associated complications. We believe these revisions improve the interpretability and clinical relevance of our findings.

**The revised discussion now reads:**

Additionally, evidence indicates that several post-stroke complications may causally contribute to delirium. A cohort study of critically ill patients with ischemic stroke showed that dysphagia increases delirium risk, partially mediated by reduced serum albumin levels [1]. Elderly patients with pneumonia are likewise more prone to delirium, with malnutrition and systemic inflammation acting as key intermediaries [2]. Experimental data further suggest that urinary tract infections can precipitate delirium via inflammatory mechanisms, while delirium itself may secondarily increase UTI risk through dehydration, urinary retention, or greater exposure to indwelling catheters, indicating a potential bidirectional relationship [3,4]. Sepsis, in particular, is a well-established trigger of acute brain dysfunction: sepsis-related systemic inflammation disrupts the blood–brain barrier and induces microcirculatory and mitochondrial dysfunction, neuroinflammation, and neurotransmitter imbalance, ultimately impairing attention and arousal networks and manifesting as delirium [5]. Collectively, these findings underscore the need for large, high-quality prospective studies to clarify causal pathways between post-stroke delirium and associated complications, thereby informing targeted prevention and management strategies.

**(Page 21, line 357-370)**

References Cited in the Response:

[1] Cheng H, Song S, Tang Y, Yuan S, Huang X, Ling Y, et al. Does ICU a-dmission dysphagia independently contribute to delirium risk in ischemic str-oke patients? Results from a cohort study. BMC Psychiatry. 2024;24(1):65. https://doi.org/10.1186/s12888-024-05520-w PMID: 38263028

[2] Liao J, Shen X, Du Z, Wang X, Miao L. Nutritional status and inflammati-on as mediators of physical performance and delirium in elderly community-acquired pneumonia patients: A retrospective cohort study. Clin Interv Aging. 2024;19:1641–1652. https://doi.org/10.2147/CIA.S483481 PMID: 39376978

[3] Winzey KD, Scott L, Moreira D, Islam TS, Bresee C, Vit JP, et al. Antimicrobial treatment ameliorates delirium-like phenotypes in a murine model of urinary tract infection. Transl Psychiatry. 2025;15(1):360. https://doi.org/10.1038/s41398-025-03624-9 PMID: 41053026

[4] Wang W, Yao W, Tang W, Li Y, Sun H, Ding W. Risk factors for urinary tract infection in geriatric hip fracture patients: a systematic review and m-eta-analysis. Front Med. 2024;11. https://doi.org/10.3389/fmed.2024.1360058 PMID: 38405191

[5] Hong Y, Chen P, Gao J, Lin Y, Chen L, Shang X. Sepsis-associated encep-halopathy: From pathophysiology to clinical management. Int Immunophar-macol. 2023;124: 110800. https://doi.org/10.1016/j.intimp.2023.110800 PMID: 37619410

**Comment 5:** *Absence of information on delirium screening and prevention methods may affect outcome interpretation.*

**Response 5:** We appreciate your important observation. We fully acknowledge that the NIS does not capture information on delirium screening protocols, diagnostic instruments, or prevention strategies implemented at individual hospitals. Consequently, we cannot exclude the possibility of under-ascertainment or differential detection of delirium across hospitals and patient groups, nor can we account for the potential confounding effect of local prevention practices on observed associations. To address this, we have explicitly acknowledged this limitation in the revised limitations.

**The revised limitations now reads:**

Moreover, because the NIS lacks information on hospital-level delirium screening tools, prevention practices, and patient-specific management strategies, delirium may be differentially detected, potentially biasing observed associations through misclassification and residual confounding.

**(Page 23, line 396-399)**

**Comment 6:** *Baseline Cognitive Status: Not reported, which is a critical factor in delirium risk.*

**Response 6:** Thank you for your insightful feedback and for emphasizing the importance of baseline cognitive status as a key risk factor for delirium. We fully agree with this observation. Regrettably, the National Inpatient Sample (NIS) database does not capture pre-hospitalization cognitive assessments, which precluded its inclusion in our analysis. To address this, we have expanded the Limitations section in the revised manuscript to explicitly discuss this constraint and its potential implications for interpreting our findings.

**The revised limitation now reads:**

Meanwhile, the lack of data in the database regarding key risk factors for delirium, notably stroke severity、pre-stroke cognitive status、drug type and frequency of administration, may have limited the precision of our estimates.

**(Page 23, line 394-396)**

**Comment 7:** *Medication Data: Missing, despite its relevance to delirium onset and progression.*

**Response 7:** Thank you for highlighting the significance of medication data in the context of delirium onset and progression. We fully concur with this observation, as pharmacological factors can profoundly influence post-stroke outcomes. In response, we have integrated available drug-related information—specifically, substance abuse—into the comorbidities analysis and expanded the Discussion section to explore its potential mechanistic roles in post-stroke delirium. Regrettably, the NIS database does not provide granular details on individual drug types or usage frequencies, which precluded a more comprehensive assessment. This constraint has been clearly articulated in the revised Limitations section.

**The revised discussion now reads:**

Notably, our findings indicate that drug abuse is an important risk factor for post-stroke delirium. Research indicates that the abuse of sedatives, in particular, may impair the function of extensive neural networks by suppressing central nervous system activity and disrupting GABAergic and adrenergic neurotransmission, thereby inducing delirium [1,2].

**(Page 17-18, line 287-290)**

References Cited in the Response:

[1] Fan Y-Y, Luo R-Y, Wang M-T, Yuan C-Y, Sun Y-Y, Jing J-Y. Mechanisms underlying delirium in patients with critical illness. Front Aging Neurosci. 2024;16. https://doi.org/10.3389/fnagi.2024.1446523 PMID: 39391586

[2] Weidmann AE, Matthíasdóttir R, Proppé GB, Tadić I, Gunnarsson PS, Jónsdóttir F. Medication Causes and Treatment of Delirium in Patients With and Without Dementia. Brain Behav. 2025;15(7):e70706. https://doi.org/10.1002/brb3.70706 PMID: 40686036

**The revised limitation now reads:**

Meanwhile, the lack of data in the database regarding key risk factors for delirium, notably stroke severity、pre-stroke cognitive status、drug type and frequency of administration, may have limited the precision of our estimates.

**(Page 23, line 394-396)**

**Comment 8:** *Include ICD Codes and this will address potential undercoding or miscoding of delirium.*

**Response 8:** Thank you for your insightful suggestion to include ICD codes, which thoughtfully addresses potential issues of undercoding or miscoding in delirium diagnoses. We concur that this addition promotes greater transparency and methodological rigor. In response, we have incorporated the relevant ICD codes into the supplementary materials (S1 File) and added a comprehensive explanation within the Methods section.

**The revised method now reads:**

Ischemic stroke、hemorrhagic stroke and post-stroke delirium were identified using ICD-9-CM and ICD-10-CM diagnostic codes (S1 File).

**(Page 6, line 112-113)**

**Comment 9:** *Clarify Stroke Subtypes and Cognitive Baseline: These are essential for accurate risk stratification.*

**Response 9:** Thank you for underscoring the critical role of stroke subtypes and baseline cognitive status in enabling accurate risk stratification for post-stroke delirium. We fully concur with this assessment and appreciate the opportunity to enhance our analysis accordingly. To address stroke subtypes, we have performed a stratified evaluation of delirium risk factors by ischemic versus hemorrhagic stroke and incorporated the detailed results into the Supplementary Materials (S2–S4 Tables in S2 File). Additionally, we have substantially revised the Discussion section to elucidate the implications of these findings for precision-based delirium management. Regrettably, the NIS database does not include pre-admission cognitive assessments, which precluded their inclusion in our study; this constraint has been explicitly highlighted in the expanded Limitations section, along with its potential impact on result interpretation.

**The revised objective now reads:(See** **S1-S4 Tables in S2 File.)**

**The revised results now reads:**

PSD occurred in 18.9% of ischemic strokes and 24.7% of hemorrhagic strokes (S1 Table in S2 File).

**(Page 8, line 142-143)**

**The revised discussion now reads:**

The findings also indicate that the incidence of delirium following hemorrhagic stroke is higher than that following ischemic stroke and the overall prevalence of post-stroke delirium, consistent with previous research [1]. Mechanisms plausibly include hematoma-associated release of blood-breakdown products and reactive oxygen species leading to robust neuroinflammation and increased exposure to ICU-related precipitating factors (e.g., mechanical ventilation, sedatives) [2-4].

**(Page 14-15, line 226-230)**

In our subgroup analyses, pre-existing hypothyroidism showed statistically significant associations with post-stroke delirium in both ischemic and hemorrhagic stroke patients. Several plausible biological mechanisms could explain why pre-existing hypothyroidism might increase the risk of delirium after ischemic stroke. Hypothyroid states promote suboptimal cerebral circulation, which increase the likelihood and severity of ischemic cerebral injury; greater ischemic burden and impaired cerebral perfusion reserve may in turn raise the brain’s vulnerability to acute cognitive disturbances such as delirium [5]. In addition, thyroid hormone deficiency is associated with pro-inflammatory and oxidative stress pathways and can impair blood–brain barrier integrity and neuronal resilience, mechanisms implicated in delirium pathogenesis [6]. Finally, hypothyroidism is linked to chronic cognitive impairment and neuropsychiatric symptoms that reduce cognitive reserve and thereby predispose patients to delirium when exposed to the acute physiological stress of ischemic stroke [7]. Therefore, close monitoring of thyroid function may serve as an effective strategy for preventing post-stroke delirium in elderly patients.

**(Page 18, line 295-307)**

Moreover, private insurance and elective admission were associated with lower odds of post-stroke delirium in the overall cohort and among ischemic stroke patients, but not in the hemorrhagic subgroup. Administrative coding (e.g., ICD) is known to have low sensitivity for detecting delirium and may differentially undercapture cases across stroke types and care settings [8]. Thus, any protective effect of private insurance and elective admission may be detectable in less-fatal ischemic strokes but obscured in the clinically more severe hemorrhagic cohort [9,10]. Residual confounding effects from baseline stroke severity and other unmeasured factors may also contribute.

**(Page 19-20, line 326-332)**

In subgroup analyses by stroke type, hospital characteristics showed divergent associations with post-stroke delirium. We observed that admission to a teaching hospital was associated with lower odds of post-stroke delirium in ischemic stroke patients, but this association was not significant in the overall cohort or the hemorrhagic subgroup. Teaching hospital and certified stroke centers typically deliver organized stroke-unit care, multidisciplinary pathways, and rapid reperfusion, and they more often implement routine delirium screening and prevention, factors that likely account for the reduced recorded delirium in the ischemic subgroup [11]. Additionally, we found that admission to an urban hospital was associated with delirium only among patients with hemorrhagic stroke. Urban centers often care for patients with a higher burden of comorbidity and adverse social determinants of health, both independently linked to delirium, which may partially explain the observed association [12]. In addition, referral and ascertainment biases, whereby severe hemorrhagic stroke cases are preferentially transferred to tertiary urban hospitals and undergo more intensive monitoring, could amplify the association in the hemorrhagic subgroup relative to ischemic stroke [12]. Thus, the higher delirium prevalence after hemorrhagic stroke seen in urban hospitals likely reflects a combination of greater baseline severity, ICU-related risks, and differential case ascertainment rather than a direct causal effect of hospital location.

**(Page 20, line 336-351)**

**The revised limitations now reads:**

Meanwhile, the lack of data in the database regarding key risk factors for delirium, notably stroke severity、pre-stroke cognitive status、drug type and frequency of administration, may have limited the precision of our estimates.

**(Page 23, line 394-396)**

References Cited in the Response:

[1] Zhang G-B, Li H-Y, Yu W-J, Ying Y-Z, Zheng D, Zhang X-K, et al. Occurrence and risk factors for post-stroke delirium: a systematic review and m-eta-analysis. Asian J Psychiatry. 2024;99:104132. https://doi.org/10.1016/j.ajp.2024.104132 PMID: 38981150

[2] Zhang G-B, Lv J-M, Yu W-J, Li H-Y, Wu L, Zhang S-L, et al. The associations of post-stroke delirium with outcomes: A systematic review and me-ta-analysis. BMC Med. 2024;22(1):470. https://doi.org/10.1186/s12916-024-03689-1 PMID: 39407191

[3] Fan Y-Y, Luo R-Y, Wang M-T, Yuan C-Y, Sun Y-Y, Jing J-Y. Mechanisms underlying delirium in patients with critical illness. Front Aging Neurosci. 2024;16. https://doi.org/10.3389/fnagi.2024.1446523 PMID: 39391586

[4] Krishnan K, Campos PB, Nguyen TN, Tan CW, Chan SL, Appleton JP, et al. Cerebral edema in intracerebral hemorrhage: pathogenesis, natural history, and potential treatments from translation to clinical trials. Front Stroke. 2023;2. https://doi.org/10.3389/fstro.2023.1256664

[5] Tian Y, Shi XQ, Shui JW, Liu XY, Bu Y, Liu Y, et al. Exploring the cau-sal factor effects of hypothyroidism on ischemic stroke: A two-sample men-delian randomization study. Front Neurol. 2024;15. https://doi.org/10.3389/fneur.2024.1322472 PMID: 38361639

[6] Zúñiga D, Balasubramanian S, Mehmood KT, Al-Baldawi S, Zúñiga Salazar G. Hypothyroidism and cardiovascular disease: a review. Cureus. 2024;16(1):e52512. https://doi.org/10.7759/cureus.52512 PMID: 38370998

[7] Sheng X, Gao J, Chen K, Zhu X, Wang Y. Hyperthyroidism, hypothyroidis-m, thyroid stimulating hormone, and dementia risk: Results from the NHA-NES 2011–2012 and mendelian randomization analysis. Front Aging Neurosci. 2024;16. https://doi.org/10.3389/fnagi.2024.1456525 PMID: 39507203

[8] Sheehan KA, Shin S, Hall E, Mak DYF, Lapointe-Shaw L, Tang T, et al. Characterizing medical patients with delirium: a cohort study comparing ICD-10 codes and a validated chart review method. PLoS One. 2024;19(5):e0302888. https://doi.org/10.1371/journal.pone.0302888 PMID: 38739670

[9] Nguyen MTH, Sakamoto Y, Maeda T, Woodward M, Anderson CS, Catiwa J, et al. Influence of socioeconomic status on functional outcomes after stroke: a systematic review and meta-analysis. J Am Heart Assoc. 2024;13(9):e033078. https://doi.org/10.1161/JAHA.123.033078 PMID: 38639361

[10] Dagli C, Huang Z, Lin C. Intracerebral hemorrhage hospitalizations and outcomes: Comparisons between institutional and national data. Intern Emerg Med. 2025;20(5):1455–1462. https://doi.org/10.1007/s11739-025-03977-5 PMID: 40437327

[11] Shen Y-C, Kim AS, Hsia RY. Treatments and patient outcomes following s-troke center expansion. JAMA Netw Open. 2024;7(11):e2444683. https://doi.org/10.1001/jamanetworkopen.2024.44683 PMID: 39535793

[12] Loggini A, Hornik J, Schwertman A, Hornik A. Rural-urban disparities in acute stroke treatments and outcomes: A propensity score-matched analysis of a nationwide sample. Cerebrovasc Dis. 2025;1-9. https://doi.org/10.1159/000546950 PMID: 40562010

**Comment 10:** *Discuss Prevention Implications: Especially for high-risk groups, to inform hospital delirium prevention programs.*

**Response 10:** Thank you for your insightful recommendation to elaborate on the prevention implications of our findings, particularly for high-risk groups, to better inform hospital delirium prevention programs. We fully agree that this addition strengthens the translational relevance of the study. In response, we have expanded the Discussion section to incorporate evidence-based strategies for delirium prevention and management in stroke patients, with a focused emphasis on high-risk subgroups, thereby offering practical guidance for clinical protocols.

**The revised discussion now reads:**

In summary, demographic factors, socioeconomic status, comorbidity burden, and post-stroke complications converge to create a high-risk state for delirium after stroke. To translate these findings into practice, we recommend routine delirium-risk assessment on admission for all older stroke patients and stratified prevention for those at greatest risk — for example, patients aged ≥80 years, with ≥3 comorbidities, a history of drug abuse, fluid and electrolyte disorders or psychoses, or post-stroke complications such as sepsis, urinary tract infection, pneumonia, or dysphagia [1,2,3]. High-risk patients should receive intensified surveillance of cognitive and psychological status, vigilance for infection, and proactive management of fluids, nutrition, and electrolytes, together with careful medication review to minimize exposure to deliriogenic drugs [2,4]. Finally, a multidisciplinary approach involving neurology, geriatrics, nursing, pharmacy, and rehabilitation is essential to implement targeted prevention and early-intervention strategies to reduce delirium incidence and improve outcomes [4,5].

**(Page 21-22, line 371-381)**

References Cited in the Response:

[1] Wischmann J, Kremer P, Hinske L, Tomasi R, Becker-Pennrich AS, Kellert L. The RAPID-score: Risk assessment and PredIction of delirium in acute stroke patients based on very early clinical parameters. Front Neurol. 2023;14:1306520. https://doi.org/10.3389/fneur.2023.1306520 PMID: 38162448

[2] Kneihsl M, Berger N, Sumerauer S, Asenbaum-Nan S, Höger FS, Gattringer T, et al. Management of delirium in acute stroke patients: A position pap-er by the austrian stroke society on prevention, diagnosis, and treatment. T-her Adv Neurol Disord. 2024;17:17562864241258788. https://doi.org/10.1177/17562864241258788 PMID: 39161955

[3] Zhang G-B, Li H-Y, Yu W-J, Ying Y-Z, Zheng D, Zhang X-K, et al. Occu-rrence and risk factors for post-stroke delirium: a systematic review and m-eta-analysis. Asian J Psychiatry. 2024;99:104132. https://doi.org/10.1016/j.ajp.2024.104132 PMID: 38981150

[4] Crone C, Fochtmann LJ, Ahmed I, Balas MC, Boland R, Escobar JI, et al. The American Psychiatric Association Practice Guideline for the Prevention and Treatment of Delirium. Am J Psychiatry. 2025;182(9):880-884. https://d oi.org/10.1176/appi.ajp.25182013 PMID: 40887950

[5] Sosnowski K, Lin F, Chaboyer W, Ranse K, Heffernan A, Mitchell M. Th-e effect of the ABCDE/ABCDEF bundle on delirium, functional outcomes, and quality of life in critically ill patients: A systematic review and meta-analysis. Int J Nurs Stud. 2023;138:104410. https://doi.org/10.1016/j.ijnurstu.2022.104410 PMID: 36577261
